# Effect of Recombinant Human Thyrotropin on the Uptake of Radioactive Iodine (^123^I) in Dogs with Thyroid Tumors

**DOI:** 10.1371/journal.pone.0050344

**Published:** 2012-11-29

**Authors:** Miguel Campos, Kathelijne Peremans, Eva Vandermeulen, Luc Duchateau, Tim Bosmans, Ingeborgh Polis, Sylvie Daminet

**Affiliations:** 1 Department of Medicine and Clinical Biology of Small Animals, Faculty of Veterinary Medicine, Ghent University, Merelbeke, Belgium; 2 Department of Veterinary Medical Imaging and Small Animal Orthopedics, Faculty of Veterinary Medicine, Ghent University, Merelbeke, Belgium; 3 Department of Comparative Physiology and Biometrics, Faculty of Veterinary Medicine, Ghent University, Merelbeke, Belgium; Consiglio Nazionale delle Ricerche (CNR), Italy

## Abstract

In humans, recombinant human thyrotropin (rhTSH) enhances radioactive iodine uptake (RAIU) in patients with differentiated thyroid cancer. No studies have been performed in veterinary medicine to optimize radioiodine treatment of thyroid cancer. The aim of this study was to evaluate the effect of rhTSH on the uptake of radioiodine-123 (^123^I) in dogs with thyroid tumors. Nine dogs with thyroid neoplasia were included in this prospective cross-over study. The dogs were divided in 2 groups. In one group, ^123^I was administered for a baseline RAIU determination in week 1. In week 2 (after a washout period of 2 weeks), these dogs received rhTSH (100 μg IV) 24 h before ^123^I injection. In the other group the order of the protocol was reversed. For each scan, the dogs received 37 MBq (1 mCi) of ^123^I intravenously (IV) and planar scintigraphy was performed after 8 and 24 h for tumor RAIU calculation. Overall, rhTSH administration caused no statistically significant change on thyroid tumor RAIU at 8 h (p = 0.89) or at 24 h (p = 0.98). A significant positive correlation was found between the effect of rhTSH on tumor 8h-RAIU and rhTSH serum concentrations at 6 h (τ = 0.68; p = 0.03), at 12 h (τ = 0.68; p = 0.03) and at 24 h (τ = 0.78; p = 0.02) after rhTSH injection. This study suggests that IV administration of 100 μg rhTSH 24 h before ^123^I has an inconsistent effect on thyroid tumor RAIU. Further studies are necessary to determine the best protocol of rhTSH administration to optimize thyroid tumor RAIU.

## Introduction

Thyroid tumors account for 10–15% of all head and neck neoplasms in dogs [1], [2]. Ninety percent of canine thyroid tumors are carcinomas and 16–38% of the patients present evidence of metastasis at the time of diagnosis [1], [3]. Surgery is the preferred treatment modality for mobile tumors, while large invasive tumors have a better prognosis with external beam radiation or radioactive iodine-131 (^131^I) therapy [1]. Two recent retrospective studies showed prolonged median survival times of 27 and 30 months following ^131^I therapy [4], [5]. Furthermore, ^131^I may be the only effective therapy against thyroid cancer metastases. In dogs, high doses of ^131^I are required and this usually implies a prolonged hospitalization period and high doses of radiation eliminated to the environment through the excreta. Use and exposure to radiation should be kept “as low as reasonably achievable” (ALARA principle) to minimize risks for patient and human health [6]. Exposure of nonthyroidal tissues to high doses of radiation may cause treatment complications such as fatal myelosuppression [4], [7]. Major limitations of ^131^I therapy include its selected effectiveness in differentiated thyroid tumors exhibiting adequate ^131^I uptake and the potential need of multiple treatments for tumor control.

In human medicine, recombinant human thyrotropin^a^ (rhTSH, Genzyme Corporation, Cambridge, ME, USA) is used to increase ^131^I uptake by normal and neoplastic thyroid tissue in the treatment and diagnostic follow-up of differentiated thyroid carcinoma [8]. In addition, the use of rhTSH before ^131^I therapy is associated with a lower whole-body exposure to radiation, limiting treatment complications [9].

Thyrotropin (TSH) binds to a membrane TSH G protein-coupled receptor on the surface of follicular thyroid cells and triggers a cascade of intracellular reactions leading to synthesis and secretion of triiodothyronine (T3), thyroxine (T4) and thyroglobulin (Tg) [10]. Prolonged TSH stimulation (>24 h) increases the expression and functionality of the Na/I symporter (NIS) and, consequently, leads to an increased uptake and organification of iodine [11], [12].

In veterinary medicine, rhTSH has been mainly used for the diagnosis of canine hypothyroidism due to the lack of specificity of the current endogenous TSH assay [13]. However, TSH receptors have already been demonstrated in canine neoplastic thyroid cells; both in primary tumors and metastases [14]. The optimization of radioiodine treatment with rhTSH may offer important clinical advantages. On one hand, by increasing the uptake^ 131^I by the thyroid tumor, rhTSH may improve ^131^I treatment efficacy and decrease the need for multiple treatments. On the other hand, rhTSH may allow a decrease of the therapeutic dosage of ^131^I, thereby improving radioprotection, limiting radiotoxicity and complying with the ALARA principle. Furthermore, ^131^I dose reduction could potentially reduce the required hospitalization period and costs.

The use of ^131^I for diagnostic imaging in clinical research has several limitations. Its half-life (8 days) makes it impractical for repeated radioactive iodine uptake (RAIU) determinations within a reasonable period. Furthermore, the emission of beta particles during the decay of ^131^I causes a higher localized radiation dose and may have a deleterious effect on the uptake of the actual ^131^I therapeutic dosage, a phenomenon named thyroid stunning [15]. Unlike ^131^I, ^123^I has a much shorter half-life (13 h), decays by emitting gamma rays and has been shown to be equal or even superior to ^131^I as a scanning agent [16]. Hence, in this study ^123^I was chosen as an imaging agent, despite its high cost. Recent pilot studies performed by our group have already investigated the use of rhTSH to optimize the uptake of radioiodine-123 (^123^I) in healthy dogs and hyperthyroid cats [17], [18]. The goal of this study was to evaluate the effect of 100 μg rhTSH, administered IV 24 h before ^123^I, on tumor RAIU in dogs with thyroid tumors.

## Materials and Methods

### Sample size

A preliminary power analysis showed that 9 patients included in a prospective cross-over study would suffice to detect a 28% increase in tumor RAIU with a power of 80% at a global significance level of 5%.

### Animals

The inclusion criteria of our study were diagnosis of thyroid neoplasia by either cytology, biopsy and/or scintigraphy and tumor uptake of ^123^I at scintigraphy. Patients where treatment was deemed urgent due to upper airway obstruction were excluded. The first nine dogs referred to the Small Animal Clinic of Ghent University that met the inclusion criteria and for which owner consent was obtained, were included. Patients were recruited between December 2007 and November 2011. Diagnosis was based on physical examination, cervical mass cytology, cervical scintigraphy and, when available, histopathology. Complete hematological and biochemical analysis, including serum total thyroxine (TT4) and TSH, were performed in all patients.

Determination of thyroid functional status was based on clinical signs, basal serum TT4 and TSH concentrations, cervical scintigraphy and TSH stimulation. Results of TSH stimulation were interpreted comparing serum TT4 concentrations at baseline and 6 h after rhTSH administration. Euthyroidism was confirmed when post-stimulation TT4 was ≥40 nmol/L and hypothyroidism was considered likely when post-stimulation TT4 concentration was <20 nmol/L [13], [19]. Intermediate results (post-stimulation TT4 between 20 and 40 nmol/L) were not observed.

All dogs were staged according to the WHO staging system for canine thyroid tumors [20]. For this purpose, cervical palpation, tridimensional measurement of the tumor, radiographs or computed tomography (CT) of the thorax, cervical and thoracic scintigraphy were performed in all patients. Cervical ultrasound was performed in 6 patients. Cervical and thoracic computed tomography was performed in 4 patients. Abdominal ultrasound was performed in 2 patients.

During the washout period, the dogs stayed at home. Diet and water source were kept unchanged during the study.

### Ethics statement

Animal care was in accordance with European guidelines and directives (EC directive 86/609/EEC for animal experiments) and the study was approved by the Ethical committee of the Faculty of Veterinary Medicine of Ghent University and by the Belgian Deontological committee (approval number EC 2010/168). Furthermore, an owner consent form was signed by all owners.

### Study design

The dogs were divided in 2 groups in a prospective cross-over study. In group A, ^123^I was administered for a baseline RAIU determination in week 1. In week 2 (after a washout period of 2 weeks), these dogs received rhTSH (100 μg IV) 24 h before ^123^I injection. In group B the order of the protocol was reversed (Table 1). For each scan, the dogs received 37 MBq (1mCi) of ^123^I IV and planar scintigraphy was subsequently performed at 8 h and 24 h for tumor RAIU calculation.

**Table 1:** Thyroid function status, tumor histopathology, primary tumor RAIU and cross-over group in 9 dogs with thyroid tumors.

Dog	Thyroid function	Histopathology	8h RAIU (%)	8h-rhTSH RAIU (%)	24h RAIU (%)	24h-rhTSH RAIU (%)	Group
1	Euthyroid	Compact carcinoma	8,5	7,8	17,4	15,7	A
2	Euthyroid	Compact carcinoma	0,2	0,8	0,2	0,6	A
3	Euthyroid	Follicular-papillary carcinoma	3,1	3,7	4,9	8,3	A
4	Hyperthyroid	Compact carcinoma	10,6	16,2	27,2	14,8	A
5	Hyperthyroid	Follicular-compact carcinoma	8,9	7,0	10,7	9,9	A
6	Euthyroid	NA	3,4	9,3	5,6	13,7	A
7	Hyperthyroid	NA	29,6	29,5	31,1	28,3	B
8	Euthyroid	C-cell carcinoma	0,7	2,0	0,9	2,8	B
9R	Hypothyroid	NA	2,1	1,7	1,8	1,6	B
9L		NA	0,8	0,4	0,7	0,5	B
		Mean ±Std Dev	6,8±8,9	7,8±9,0	10,0±11,4	9,6±8,9	

The mean and standard deviation of the 8 h- and 24 h-RAIU with and without rhTSH stimulation are given.

NA: not available; R: right; L: left.

### Blood samples

Blood samples were taken for serial measurements of TT4 and rhTSH serum concentrations in the week rhTSH was administered. Blood was collected by jugular venipuncture at baseline, 6, 12, 24 and 48 h after rhTSH injection. Blood was centrifuged and the serum was stored for at least 3 weeks at −20°C to reach sufficient decay of radioactivity to be analyzed.

The TT4 serum concentration was determined with a commercially available solid-phase, chemiluminescent competitive immunoassay (IMMULITE 2000 Canine Total T4, Siemens, Deerfield, IL, USA) previously validated in dogs, and the reference range used was 6.45–43.86 nmol/L [21]. Basal TSH serum concentration was determined with a commercially available solid-phase, two-site chemiluminescent immunometric assay (IMMULITE 2000 Canine TSH, Siemens, Deerfield, IL, USA) previously validated in dogs, and the reference range used was <0.5 ng/mL [21].

rhTSH serum concentrations were measured with a commercially available chemiluminescent microparticle immunoassay for human TSH determination in an immunoassay analyzer (Abbott ARCHITECT *i*2000SR, Abbott Laboratories, Abbott Park, IL, USA) [22].

### Recombinant human TSH

Each vial of 900 µg of rhTSH was reconstituted with 4.5 mL of sterile water (200 µg/mL). Individual doses of 100 µg of freshly reconstituted rhTSH were prepared in 1 mL plastic syringes with needle and rubber caps and were stored frozen at −20°C for a maximum of 12 weeks [13], [23]. For TSH stimulation, frozen rhTSH was thawed at room temperature a few minutes before administration.

### RAIU

Each dog received 37 MBq (1 mCi) of ^123^I IV. The injected activity of ^123^I was calculated by subtracting the activity of the empty syringe from the activity of the full syringe both measured in a dose calibrator. To determine the tumor/metastases RAIU, a static planar ventrodorsal image was obtained with a one head γ-camera (Toshiba GCA 901) using a low-energy high resolution collimator with the dog in sternal recumbency under general anesthesia. General anesthesia was induced with propofol and maintained with isoflurane vaporized in oxygen using a rebreathing system. Data were acquired during 5 minutes for the 8 h-RAIU and 10 minutes for the 24 h-RAIU on a 128×128 matrix. A syringe with a known amount of radioactivity (2.5±1.6 MBq) was placed next to the animal and served as the standard activity necessary to calculate the RAIU. Regions of interest (ROI) were manually drawn over the primary tumor/metastases and over the activity of the standard [24]. In order to correct for background activity a ROI with the same dimensions as the ROI over the tumor was drawn over an area close to, but not overlapping, the thyroid tumor (soft tissue background correction) and another ROI with the same dimensions of the ROI over the standard was placed outside the dog (room background correction). The total number of counts in each ROI was recorded and transformed to counts per minute (cpm) for RAIU calculation, yielding cpm_tumor_, cpm_standard_, cpm_background_ and cpm_room_. These ROI's were placed on one day and by the same person (EV). RAIU was calculated as a percentage of the administered dose of ^123^I corrected for physical decay and background activity using the following formula:
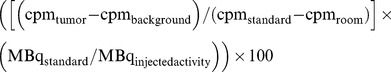



### Statistical analysis

Data were analyzed with SAS version 9.1 (SAS, Cary, North Carolina, USA). The effect of rhTSH administration on RAIU was analyzed with a mixed model with period, treatment, time and the interaction between treatment and time as categorical fixed effects and dog and the period by dog interaction as random effects. Comparisons were based on the F-test at a global significance level of 5%, using Tukey's procedure for multiple comparisons.

The change of TT4 serum concentration from baseline to 6 h was analyzed using a mixed model with dog as random effect and time as a categorical fixed effect.

Possible associations between the effect of rhTSH on tumor RAIU (at either 8 h or 24 h) and rhTSH serum concentration at all time points and results of TSH stimulation were evaluated with the Kendall's τ correlation coefficient.

The association between the effect of rhTSH on tumor RAIU (at either 8 h or 24 h) and thyroid function status (euthyroid vs hyperthyroid) was evaluated on a data set excluding the one hypothyroid patient that was in the data set. The analysis was based on a mixed model with period, treatment, time, status and the interactions between treatment, time and status as categorical fixed effects and the dog and the period by dog interaction as random effects.

## Results

Two mixed breed dogs, 1 medium-sized Poodle, 1 American Staffordshire Terrier, 1 German Longhaired Pointer, 1 Jack Russell Terrier, 1 Bearded Collie, 1 Belgian Shepherd Malinois and 1 Beagle were included in this study. Six dogs were males, 3 were females, mean age was 9.5 years (range 6–12 years).

Six dogs were diagnosed with unilateral thyroid tumors, 1 dog had bilateral thyroid tumors and 2 dogs had ectopic tumors. Three patients were diagnosed with thoracic metastases visible at scintigraphy (n = 2) and radiographs (n = 1). Histopathology was performed in 6 patients. The only patient for which cytology or histopathology were not performed was diagnosed with an ectopic thyroid tumor clearly visible at scintigraphy. Furthermore, the dog had clinical hyperthyroidism.

Five dogs were euthyroid, 3 dogs were hyperthyroid and 1 dog was hypothyroid. All patients presented for a palpable cervical mass. Additionally, the 3 hyperthyroid dogs presented with PU/PD and weight loss; two of these also presented polyphagia. The hypothyroid dog had been diagnosed several years prior to referral and was being treated with levothyroxine supplementation. One dog had been previously diagnosed and treated for hypothyroidism but was reclassified as euthyroid based on basal TT4 serum concentrations, cervical scintigraphy and TSH stimulation results at the moment of inclusion in our study. In both cases, levothyroxine supplementation was interrupted for at least 3 days before the study week. Four patients were diagnosed with stage II, 2 patients with stage III and 3 patients with stage IV thyroid cancer.

**Figure 1:**
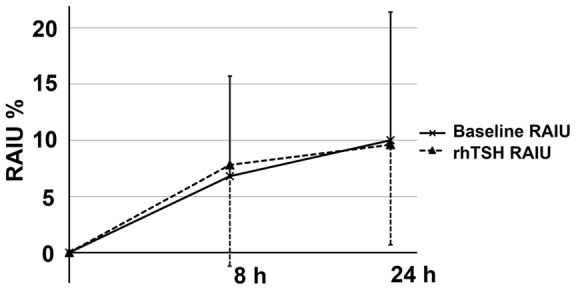
Primary thyroid tumor RAIU in 9 dogs with thyroid tumors. The mean and standard deviation of the 8 h- and 24 h-RAIU with and without rhTSH stimulation are given.

The results of thyroid function status, tumor histopathology and tumor RAIU determination of each dog are summarized in Table 1. After rhTSH administration, the 8 h-RAIU increased in 5 tumors and the 24 h-RAIU increased in 4 tumors. Overall, rhTSH caused no statistically significant change on primary thyroid tumor RAIU at 8 h (p = 0.89) or at 24 h (p = 0.98) (Figure 1). The RAIU of thoracic metastases could only be evaluated in 2 of the 3 patients with stage IV disease. After rhTSH administration, the 8 h- and 24 h-RAIU increased in 1 thoracic metastasis (Table 2).

**Table 2:** Thyroid function status, metastases RAIU and cross-over group in 2 dogs with thoracic metastases.

Dog	Thyroid function	8h RAIU (%)	8h-rhTSH RAIU (%)	24h RAIU (%)	24h-rhTSH RAIU (%)	Group
3 – thorax	Euthyroid	0.05	0.15	0.04	0.09	A
8 – thorax	Euthyroid	0.03	0.01	0.06	0.01	B

TT4 and rhTSH serum concentrations were measured immediately before and followed up after rhTSH injection in 4 euthyroid dogs, 1 hyperthyroid dog and 1 hypothyroid dog. The 4 euthyroid patients showed a significant increase in TT4 serum concentrations 6 h after rhTSH injection compared to baseline (p = 0.01) (Table 3). The hyperthyroid dog and the hypothyroid dog did not show meaningful changes in serum TT4 concentrations at any time point.

**Table 3:** rhTSH and TT4 serum concentrations in dogs with thyroid tumors before and after injection of 100 μg rhTSH IV.

Time point	rhTSH (mIU/L)	TT4 (nmol/L)
Baseline	0 (0)	24.2 (6.45)
6 h	26.97 (4.47)	50.97 (7.03)
12h	9.52 (1.45)	35.79 (10.47)
24 h	3.30 (0.63)	25.83 (9.1)
48 h	0.77 (0.27)	22.28 (6.94)

The mean values (standard deviation) of rhTSH serum concentrations of 6 dogs and TT4 serum concentrations of 4 euthyroid dogs with thyroid tumors are given for each time point.

A significant positive correlation was found between the effect of rhTSH on tumor 8 h-RAIU and rhTSH serum concentrations at 6 h (τ = 0.68; p = 0.03), at 12 h (τ = 0.68; p = 0.03) and at 24 h (τ = 0.78; p = 0.02) after rhTSH injection. When tumor metastases were included in the analysis, a significant positive correlation was also detected between the effect of rhTSH on 24 h-RAIU and rhTSH serum concentrations at 6 h (τ = 0.59; p = 0.04), 24 h (τ = 0.59; p = 0.04) and 48 h (τ = 0.67; p = 0.02). No significant correlation was found between the change in TT4 serum concentrations from baseline to 6 h and the effect of rhTSH on tumor RAIU, at either 8 h (p = 0.54) or 24 h (p = 0.13).

Hyperthyroid dogs had significantly higher RAIU values than euthyroid dogs (p = 0.01). Furthermore, the effect of the rhTSH on 24 h-RAIU differed significantly between euthyroid and hyperthyroid dogs (p = 0.02) but not at 8 h (p = 0.98). In euthyroid dogs, the 24 h-RAIU increased from 5.8%, at baseline, to 8.2%, after rhTSH. In hyperthyroid dogs, the 24 h-RAIU decreased from 23%, at baseline, to 17.7%, after rhTSH.

No adverse effects were observed following the administration of rhTSH.

## Discussion

Thyroid cancer is the most common endocrine neoplasia in dogs and, as in humans, these tumors are mainly of follicular cell origin [1]. In humans, rhTSH stimulates the uptake of ^131^I in patients with differentiated thyroid carcinoma and can be used to improve the efficacy of ^131^I therapy [25]. Additionally, the use of rhTSH before ^131^I therapy is associated with a lower ^131^I effective half-life and, consequentely, lower exposure of blood and the whole-body to radiation. This limits radiotoxicity without compromising the efficacy of the treatment [9]. The obvious benefits of the use of rhTSH to optimize the treatment of human thyroid cancer with ^131^I provides an interesting perspective for the optimization of ^131^I therapy of canine thyroid tumors. Increased ^131^I uptake by canine thyroid tumors may improve treatment efficacy and decrease the need for multiple treatments; reduced blood exposure to radiation may limit myelossupression; ^131^I dose reduction could improve radioprotection, reduce the hospitalization period and costs. This is the first study to evaluate the effect of rhTSH on radioiodine uptake in dogs with thyroid tumors.

Overall, no significant effect of rhTSH on tumor RAIU was observed with our protocol. These results are in agreement with the results of a recent pilot study performed by our group in healthy Beagles [17]. In that study, rhTSH (100 μg IV, administered 24 h or 48 h before ^123^I) also did not cause a significant change in thyroid RAIU. Earlier reports have suggested the potential of exogenous TSH to increase thyroid RAIU in dogs [26], [27], [28]. However, in these studies the effect of TSH stimulation on thyroid ^131^I uptake was described in a small number of healthy and hypophysectomized dogs and no statistical analysis was performed. In healthy humans, TSH stimulation with a protocol similar to the one used in this study was shown to approximately double thyroid RAIU [29].

The inconsistent effect of rhTSH on thyroid tumor RAIU observed in our study raises important issues regarding the dosage, the route and timing of rhTSH administration. The significant increase in TT4 serum concentrations 6 h after rhTSH injection, observed in euthyroid patients, was expected and confirmed the biological activity of rhTSH. The observed correlation between the effect of rhTSH on tumor RAIU and rhTSH serum concentrations suggests that higher plasma concentrations of rhTSH may allow an increased uptake of ^123^I by thyroid tumors. The plasma concentration of rhTSH at different time points is mainly related to the dose administered. It is possible that doses higher than 100 μg may induce a more consistent increase of thyroid tumor RAIU. In humans, high plasma concentrations of TSH (>30 mIU/mL) are deemed necessary to stimulate sodium-iodide symporters to concentrate iodine, in normal and neoplastic thyroid tissue. Hence, high doses of rhTSH (2×900 μg IM 24 h apart) are administered 24 h before ^131^I injection for diagnostic follow-up and treatment of differentiated thyroid cancer. Nevertheless, the optimal magnitude of TSH elevation is unknown and differs among patients [30]. It is interesting to note that, in our study, all patients with rhTSH serum concentrations >30 mIU/mL 6 h after rhTSH injection experienced an increase in thyroid tumor RAIU after rhTSH. The administration of rhTSH doses similar to those given to humans with thyroid cancer is not realistic in the veterinary clinical setting given the high cost of rhTSH. Furthermore, studies in humans with multinodular goiter have demonstrated that rhTSH doses as low as 5 or 100 μg suffice to effectively increase thyroid RAIU [31]. In our study, the dosage of 100 μg was chosen because this dose is considered appropriate for a functional stimulation of the thyroid gland in most dogs [13]. Further studies are necessary to determine the effect of higher doses of rhTSH on thyroid tumor RAIU in dogs.

An important factor influencing the pharmacokinetics of rhTSH is the route of administration. In our study, the IV route was chosen to maximize bioavailability. Although there are no reports arguing that the IM route is preferable, it is possible that if rhTSH is administered IM, such as in humans, its clearance is slower allowing a longer stimulation of the thyroid cells and possibly increasing tumor RAIU more consistently.

In our study, rhTSH was administered 24 h before ^123^I because studies on FRTL-5 cells (Fischer rat thyroid cell line) revealed that 12 to 24 h are needed before TSH stimulates accumulation of iodine in thyroid cells and because this is considered the optimal timing to increase thyroid RAIU in humans [11], [32]. The optimal timing of rhTSH administration to increase thyroid RAIU in dogs with thyroid tumors is currently unknown.

The inconsistent effect of rhTSH on thyroid tumor RAIU may not be related to the protocol of rhTSH administration but rather to intrinsic properties of the neoplastic thyroid tissue. It has been shown that the concentration and affinity of TSH receptors in neoplastic canine thyroid cells is variable [14]. Indeed, 8 of 22 primary canine thyroid tumors were shown to have fewer TSH-binding sites than the lowest value observed in normal thyroid tissues. That study suggested that TSH receptor concentration could be related to the functional variability of thyroid neoplasms. Likewise, studies in humans have demonstrated that the expression of TSH-receptor mRNA may be decreased in thyroid carcinomas [33]. Additionally, *in vitro* studies have revealed that TSH unresponsiveness in human thyroid carcinomas can also be related to defects in TSH signal transduction or errors in iodine transport [34], [35].

As expected, our study showed that hyperthyroid dogs had significantly higher RAIU values than euthyroid dogs. In hyperthyroidism, increased thyroid function enhances iodine trapping and organification. Hence, dogs with thyroid tumors and hyperthyroidism frequently present high tumoral ^131^I uptake and are often ideal candidates for ^131^I therapy [36]. The significantly different effect of rhTSH on thyroid RAIU between euthyroid and hyperthyroid patients was an interesting finding. In a study of 55 dogs with thyroid tumors, dogs with evidence of autonomous hyperfunction of the goiter had an increased thyroidal iodine turn-over [28]. It is possible that a positive effect of rhTSH on tumor RAIU occurs sooner in hyperthyroid patients and was, therefore, not observed with our protocol (RAIU determination 8 h and 24 h after ^123^I injection). On the other hand, the lack of effect of rhTSH in hyperthyroid patients may be caused by decreased thyroid functional reserve. This seems, however, less likely because in hyperthyroid cats and in humans with toxic nodular goiter (a condition characterized by nodular enlargement of the thyroid gland and hyperthyroidism), a significant increase in thyroid RAIU is observed after rhTSH administration [18], [37].

The administration of rhTSH to patients with thyroid carcinoma raises important safety issues. In humans, rhTSH causes expansion of primary thyroid tumors and thyroid tumor metastases [38], [39]. Therefore, rhTSH should be used carefully in patients with large thyroid tumors or central nervous system, spinal, lung or bone metastases. A pilot study performed in healthy Beagles showed no effect of rhTSH on thyroid gland volume [40]. Likewise, no adverse effects of rhTSH were observed during our study.

All thyroid tumors for which histopathology was available were malignant and 5 of the 6 tumors were of follicular cell origin. This was expected as 90% of canine thyroid tumors are malignant and only patients with ^123^I uptake were included [1]. In humans and dogs, malignant thyroid tumors are predominantly of follicular cell origin, but in humans only 8.1–14.8% of all thyroid nodules are malignant [41]. Another important difference resides in the predominant histologic types. In dogs, thyroid carcinomas are predominantly mixed follicular-compact, while in humans 80% of thyroid malignancies are of papillary type which is rare in dogs [42], [43]. Undifferentiated carcinomas are relatively uncommon in both species, accounting for 2% of thyroid malignancies in humans and 12% in dogs.

One dog with a C-cell carcinoma presented ^123^I uptake by the primary tumor and thoracic metastases. To the authors knowledge, there is only one previous report in veterinary medicine and very limited reports in human medicine of medullary carcinomas exhibiting iodine uptake [36], [44], [45]. The mechanism underlying the ability of medullary carcinoma cells to trap iodine remains unclear.

In conclusion, our study shows that 100 μg rhTSH administered IV 24 h before ^123^I has no significant effect on thyroid tumor RAIU in dogs. The detected correlation between increased tumor RAIU and rhTSH serum concentrations attained after injection suggests that higher dosages of rhTSH may be necessary. Further studies are needed to determine the optimal protocol of rhTSH administration to increase thyroid tumor RAIU in dogs.
